# Supplementary material for: People are more error-prone after committing an error
Source: Nat Commun. 2024 Jul 30;15:6422. doi: 10.1038/s41467-024-50547-y (PMC11289479; doi:10.1038/s41467-024-50547-y)
Supplement: Supplementary file 3 — Reporting Summary [file 41467_2024_50547_MOESM3_ESM.pdf]

nature portfolio

Corresponding author(s): Taraz Lee

Last updated by author(s): Jun 18, 2024

Reporting Summary

Nature Portfolio wishes to improve the reproducibility of the work that we publish. This form provides structure for consistency and transparency in reporting. For further information on Nature Portfolio policies, see our Editorial Policies and the Editorial Policy Checklist.

Statistics

For all statistical analyses, confirm that the following items are present in the figure legend, table legend, main text, or Methods section.

n/a	Confirmed	
☐	☒	The exact sample size (*n*) for each experimental group/condition, given as a discrete number and unit of measurement
☐	☒	A statement on whether measurements were taken from distinct samples or whether the same sample was measured repeatedly
☐	☒	The statistical test(s) used AND whether they are one- or two-sided<br>*Only common tests should be described solely by name; describe more complex techniques in the Methods section.*
☐	☒	A description of all covariates tested
☐	☒	A description of any assumptions or corrections, such as tests of normality and adjustment for multiple comparisons
☐	☒	A full description of the statistical parameters including central tendency (e.g. means) or other basic estimates (e.g. regression coefficient) AND variation (e.g. standard deviation) or associated estimates of uncertainty (e.g. confidence intervals)
☐	☒	For null hypothesis testing, the test statistic (e.g. *F*, *t*, *r*) with confidence intervals, effect sizes, degrees of freedom and *P* value noted<br>*Give P values as exact values whenever suitable.*
☐	☒	For Bayesian analysis, information on the choice of priors and Markov chain Monte Carlo settings
☐	☒	For hierarchical and complex designs, identification of the appropriate level for tests and full reporting of outcomes
☒	☐	Estimates of effect sizes (e.g. Cohen's *d*, Pearson's *r*), indicating how they were calculated

Our web collection on statistics for biologists contains articles on many of the points above.

Software and code

Policy information about availability of computer code

Data collection	Psychopy (Peirce, J. W., Gray, J. R., Simpson, S., MacAskill, M. R., Höchenberger, R., Sogo, H., Kastman, E., Lindeløv, J. (2019). PsychoPy2: experiments in behavior made easy. Behavior Research Methods. 10.3758/s13428-018-01193-y)
Data analysis	Stan (Carpenter, B., Gelman, A., Hoffman, M. D., Lee, D., Goodrich, B., Betancourt, M., Brubaker, M., Guo, J., Li, P., & Riddell, A. (2017). Stan: A Probabilistic Programming Language. Journal of Statistical Software, 76(1). https://doi.org/10.18637/jss.v076.i01)<br>brms (Bürkner, P.-C. (2017). brms: An R Package for Bayesian Multilevel Models Using Stan. Journal of Statistical Software, 80(1). https://doi.org/10.18637/jss.v080.i01)<br><br>The datasets generated and analyzed by the studies presented here are available in an Open Science Framework repository: doi.org/10.17605/OSF.IO/U7SZR<br><br>Computer code used for the analyses and figures can be found at https://github.com/adkinsty/post_error_followup.

For manuscripts utilizing custom algorithms or software that are central to the research but not yet described in published literature, software must be made available to editors and reviewers. We strongly encourage code deposition in a community repository (e.g. GitHub). See the Nature Portfolio guidelines for submitting code & software for further information.

Data

Policy information about availability of data

All manuscripts must include a data availability statement. This statement should provide the following information, where applicable:

- Accession codes, unique identifiers, or web links for publicly available datasets
- A description of any restrictions on data availability
- For clinical datasets or third party data, please ensure that the statement adheres to our policy

The datasets generated and analyzed by the studies presented here are available in an Open Science Framework repository: doi.org/10.17605/OSF.IO/U7SZR

Research involving human participants, their data, or biological material

Policy information about studies with human participants or human data. See also policy information about sex, gender (identity/presentation), and sexual orientation and race, ethnicity and racism.

Reporting on sex and gender	We did not perform sex or gender based analyses. Our samples were split evenly (~50%) between men and women (self-identified) and we had no explicit hypotheses or reason to suspect there would be sex/gender differences.
Reporting on race, ethnicity, or other socially relevant groupings	We did not use any of these variables
Population characteristics	In Experiment 1 there were 46 participants (24 female) with a mean age of 33 years old. In Experiment 2 there were 37 participants (33 female) with a mean age of 27 years old. In experiment 3 there were 47 participants (24 female, 1 declined to answer) with a mean age of 29 years old. In Experiment 4, there were 46 participants (22 female, 3 declined to answer) with a mean age of 32 years old.
Recruitment	We recruited online through the Prolific platform. Although the Prolific subject pool is more representative of the US population than the local University of Michigan community, there may be a self-selection bias in terms of people who would opt for online experiments. We don't anticipate this has a significant impact on the results presented here.
Ethics oversight	University of Michigan Health Sciences and Behavioral Sciences Institutional Review Board

Note that full information on the approval of the study protocol must also be provided in the manuscript.

Field-specific reporting

Please select the one below that is the best fit for your research. If you are not sure, read the appropriate sections before making your selection.

☐ Life sciences ☒ Behavioural & social sciences ☐ Ecological, evolutionary & environmental sciences

For a reference copy of the document with all sections, see nature.com/documents/nr-reporting-summary-flat.pdf

Behavioural & social sciences study design

All studies must disclose on these points even when the disclosure is negative.

Study description	This is a quantitative cognitive psychology (psychophysics) study.
Research sample	Participants were recruited online using the online platform Prolific with the following inclusion criteria: US or Canadian nationality, fluent English speaker, approval rate >95%. In Experiment 1 there were 46 participants (24 female) with a mean age of 33 years old. In Experiment 2 there were 37 participants (33 female) with a mean age of 27 years old. In experiment 3 there were 47 participants (24 female, 1 declined to answer) with a mean age of 29 years old. In Experiment 4, there were 46 participants (22 female, 3 declined to answer) with a mean age of 32 years old.
Sampling strategy	We did not perform a formal statistical power analysis for our first experiment and the sample size was a sample of convenience that exceeded sample size typically seen in the published post-error slowing literature. As the rest of the experiments were essentially conceptual replications of the first result, we chose to maintain the same sample size as our results were quite reliable and robust.
Data collection	Data was collected online through the Pavlovia/Psychopy platform
Timing	Data collection occurred September, 2021 through December, 2021
Data exclusions	We did not exclude any participant's data from the analyses
Non-participation	No participant dropped out of the study

Randomization	N/A. All our analyses were within-subject and all subjects were exposed to all experimental conditions.

Reporting for specific materials, systems and methods

We require information from authors about some types of materials, experimental systems and methods used in many studies. Here, indicate whether each material, system or method listed is relevant to your study. If you are not sure if a list item applies to your research, read the appropriate section before selecting a response.

Materials & experimental systems

n/a	Involved in the study
☒	☐ Antibodies
☒	☐ Eukaryotic cell lines
☒	☐ Palaeontology and archaeology
☒	☐ Animals and other organisms
☒	☐ Clinical data
☒	☐ Dual use research of concern
☒	☐ Plants

Methods

n/a	Involved in the study
☒	☐ ChIP-seq
☒	☐ Flow cytometry
☒	☐ MRI-based neuroimaging

Plants

Seed stocks	*Report on the source of all seed stocks or other plant material used. If applicable, state the seed stock centre and catalogue number. If plant specimens were collected from the field, describe the collection location, date and sampling procedures.*
Novel plant genotypes	*Describe the methods by which all novel plant genotypes were produced. This includes those generated by transgenic approaches, gene editing, chemical/radiation-based mutagenesis and hybridization. For transgenic lines, describe the transformation method, the number of independent lines analyzed and the generation upon which experiments were performed. For gene-edited lines, describe the editor used, the endogenous sequence targeted for editing, the targeting guide RNA sequence (if applicable) and how the editor was applied.*
Authentication	*Describe any authentication procedures for each seed stock used or novel genotype generated. Describe any experiments used to assess the effect of a mutation and, where applicable, how potential secondary effects (e.g. second site T-DNA insertions, mosiacism, off-target gene editing) were examined.*
